# Npas4 Expression in Two Experimental Models of the Barrel Cortex Plasticity

**DOI:** 10.1155/2015/175701

**Published:** 2015-02-16

**Authors:** Aleksandra Kaliszewska, Malgorzata Kossut

**Affiliations:** Department of Molecular and Cellular Neurobiology, Nencki Institute, 3 Pasteur Street, 02-093 Warsaw, Poland

## Abstract

Npas4 has recently been identified as an important factor in brain plasticity, particularly in mechanisms of inhibitory control. Little is known about Npas4 expression in terms of cortical plasticity. In the present study expressions of Npas4 and the archetypal immediate early gene (IEG) c-Fos were investigated in the barrel cortex of mice after sensory deprivation (sparing one row of whiskers for 7 days) or sensory conditioning (pairing stimulation of one row of whiskers with aversive stimulus). Laser microdissection of individual barrel rows allowed for analysis of IEGs expression precisely in deprived and nondeprived barrels (in deprivation study) or stimulated and nonstimulated barrels (in conditioning study). Cortex activation by sensory conditioning was found to upregulate the expression of both Npas4 and c-Fos. Reorganization of cortical circuits triggered by removal of selected rows of whiskers strongly affected c-Fos but not Npas4 expression. We hypothesize that increased inhibitory synaptogenesis observed previously after conditioning may be mediated by Npas4 expression.

## 1. Introduction

Npas4 has been recognized as a brain-specific transcription factor [1, 2], important for structural and functional neuronal plasticity. Lin et al. [3] identified it as an element of the program controlling inhibitory synapse development and plasticity. They postulated that Npas4 induction in response to increased excitatory input acts to reduce activity levels and therefore may serve to maintain the homeostatic balance between excitation and inhibition. In accordance with this hypothesis, Sim et al. [4] found that Npas4 is required for activity-dependent increases in GABAergic input on dentate gyrus granule cells and that Npas4 signaling within individual neurons in the dentate gyrus is necessary to trigger activity-dependent changes in dendritic morphology. Findings of Bloodgood et al. [5] indicate that in pyramidal neurons of hippocampus of mice kept in enriched environment Npas4 promotes an increase in the number of inhibitory synapses on the cell soma and a decrease in the number of inhibitory synapses on the apical dendrites. Spiegel et al. [6] demonstrated that Npas4 promotes the development of excitatory, but not inhibitory, synapses onto somatostatin interneurons. This opposite impact of Npas4 on connectivity of excitatory and inhibitory neurons is explained by its ability to activate cell-type specific transcriptional programs. It is hypothesized that Npas4 works as a part of homeostatic mechanism to restrict excitation within neuronal circuits. Npas4 mediates structural plasticity by controlling neurite outgrowth in vitro [7], dendrite polarization within the barrel cortex during development [8], and sensory-driven changes in density of spines in olfactory bulb granule cells [9]. Npas4 is capable of regulating transcription of drebrin [2], which is involved in structural changes in dendritic spines [10].

Studies of Npas4 expression in learning and memory mainly concern limbic structures. It was implicated in context learning and long term-contextual memory [11] and amygdala-dependent fear conditioning [12]. The role of Npas4 in cortical plasticity has just recently started to be elucidated. Npas4 was reported to play a role in mediating the reinstatement of ocular dominance plasticity in the visual cortex of adult rats following fluoxetine treatment [13]. Here we characterized Npas4 expression in adult mouse barrel cortex using two models of plasticity: sensory deprivation and sensory conditioning.

Local elimination of excitatory input to deprived cortical barrels disrupts the equilibrium between excitation and inhibition, which leads to modifications in functional activation and anatomical rewiring of cortical sensory areas. Functional representation of spared inputs starts to expand to deprived areas [14–19]. This is accompanied by large-sale structural changes, such as axonal retraction and sprouting [20–23] and dendritic reorganization [24, 25], and also by more subtle changes involving spines and synapses, with decreased density of inhibitory synapse in deprived barrels [26–31]. Classical conditioning involving unilateral stimulation of row B vibrissae paired with tail shock results in behavioral changes (increased immobility), expansion of functional cortical representation of the row of vibrissae stimulated during conditioning, and increased density of inhibitory synapses on spines in barrels that represent the stimulated vibrissae [32, 33]. As Npas4 is implicated in structural plasticity, in the present study we aimed to determine how Npas4 expression in the barrel cortex is influenced by sensory conditioning and sensory deprivation.

## 2. Methods

### 2.1. Deprivation and Somatosensory Stimulation

**Figure 1:**
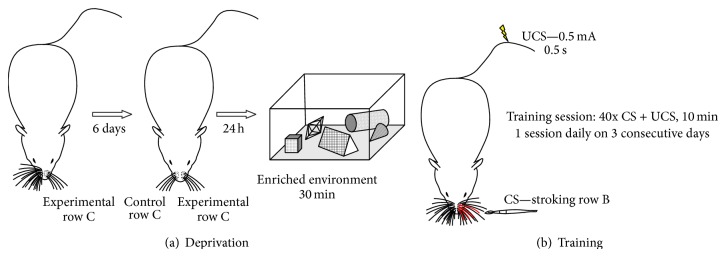
(a) Scheme of the deprivation procedure. All whiskers on one side of the snout except for row C are removed. After 6 days the same deprivation procedure is applied to the other side of the snout and the mouse is left with both C rows intact. After 24 h the animal explores a stimulatory cage for 30 minutes and is then immediately killed. (b) Scheme of the training procedure. Row B of whiskers on the left side of the snout is stroked (CS). The CS lasts for 9 s. During the last second an electric shock (0.5 mA for 0.5 s, UCS) is delivered and coterminates with the CS. A single training session lasts for 10 minutes and encompasses 40 CS and UCS pairings. The animal is subjected to one session daily for three consecutive days. Following the last training session, the mouse is placed in its home cage for 20 minutes to allow increase in Npas4 expression and is then killed.

Six male C57BL/6 mice aged 8-9 weeks were used in the deprivation experiment. The mice were reared in a 12 : 12 light/dark cycle in standard cages and had ad libitum access to water and food. All experimental procedures were approved by the First Ethical Commission in Warsaw, Poland, and were in accordance with the European Communities Council Directive of 24 November 1986 (86/609/EEC). Mice were deprived under short (2-3 minutes) isoflurane anesthesia by plucking out all whiskers except row C on one side of the snout (referred to as experimental side later in the text, Figure 1(a)). This experimental model of sensory deprivation leaves the centrally situated row C of vibrissae intact, with symmetrical space for remodeling of the cortex in the medial and lateral direction. Regrowing vibrissae were removed every second day. On the 6th day (24 hours before the experiment) the other side of the snout (control side) was subjected to the same deprivation procedure, so that mice were left with intact row C whiskers on both sides. Animals were deprived 24 hours prior to exploration of the stimulatory cage to avoid increase in Npas4 and c-Fos expression induced by whisker plucking. Mice were allowed to explore the stimulatory cage on the 7th day. The walls and floor were made of bars, and the cage was equipped with objects of different shapes and textures: mouse wheels, plastic toys, maze-like constructions, and pieces of Styrofoam. Animals were placed in the cage and left for 30 min in a room without illumination to promote sensory stimulation. Next, they were killed by cervical dislocation. The brains were dissected out, and cortices were flattened [34] and frozen in −70°C isopentane.

### 2.2. Training Procedure

29 male Swiss albino mice aged 8-9 weeks were used in training procedure. Prior to behavioral training, mice were habituated to a neck restraint for 10 min a day, 5 days a week, for 2-3 weeks. Animals were placed in separate home cages a few days before onset of the training. During training, row B whiskers on the left side of the snout were stroked in the posterior to anterior direction (CS) with a fine handheld brush (Figure 1(b)). The CS lasted for 9 s. During the last second, an aversive UCS (a mild electric shock of 0.5 mA for 0.5 s applied to the tail) was delivered and coterminated with the CS. This trial was repeated after a 6 s interval and the routine was continued for 10 min. Training encompassed three training sessions on three consecutive days. Following the last training session, animals were placed in their home cages for 20 minutes to allow for increase in Npas4 expression and then killed by cervical dislocation. The brains were dissected out, and cortices were flattened [34] and frozen in −70°C isopentane.

The following experimental groups were examined:

- CS + UCS (*n* = 9): pairing whisker stimulation with tail shock;
- PSEUDO (pseudoconditioned, *n* = 7): unpaired application of the same number of CS and UCS as during conditioning;
- CS only (*n* = 8): sessions of whisker stroking;
- naïve – (*n* = 5): unstimulated controls in neck restraining apparatus.

### 2.3. Laser Microdissection

**Figure 2:**
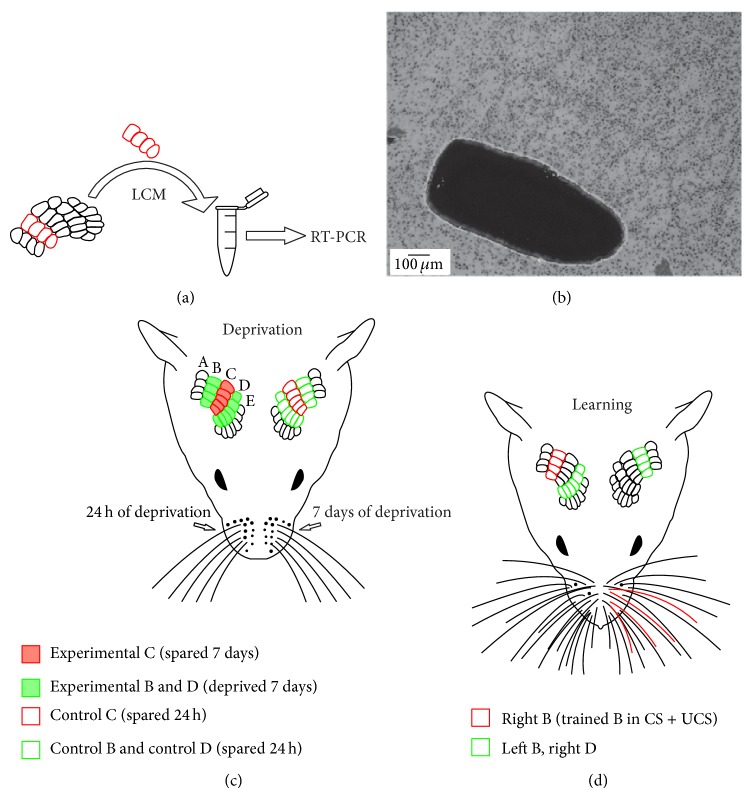
(a) Single rows of barrels are microdissected from Nissl-stained sections. RNA from dissected tissue is extracted and processed for RT-PCR. (b) Nissl-stained section with row B dissected out. Neighbouring row C is left intact. ((c), (d)) Rows of barrels microdissected in deprivation (C) and sensory conditioning (D) experiment. Microdissected barrels are colored in green and red. Legend below the scheme explains terminology used in the text for description of particular rows.

Flattened cortices were cryosectioned (16 *μ*m) tangentially to the surface. Slices from layer IV were mounted on membrane slides (MembraneSlide 1.0 PEN, Carl Zeiss MicroImaging GmbH) and stored at −70°C until further usage. Immediately prior to the microdissection, slices were subjected to modified Nissl staining using Arcturus HistoGene Staining Solution (KIT0401, Life Technologies), which preserves nucleic acid integrity. Sections from both hemispheres of the animal were processed together. Isolation of tissue was performed using the ArcturusXT LCM System equipped with a Nikon Eclipse Ti-E microscope (Figures 2(a) and 2(b)). Microdissected tissue was collected on CapSure Macro LCM Caps (LCM0211, Life Technologies). In slices from mice used in the conditioning experiment, rows B and D from the right hemisphere were dissected and from the left hemisphere only row B was dissected (Figure 2(d)). Row B from the right hemisphere in the CS + UCS group is referred to as the trained row of whiskers. In slices from deprived mice, rows B, C, and D from both hemispheres were microdissected (Figure 2(c)). Row C was collected on a separate membrane and rows B and D were collected on the same membrane. In further steps, tissue from rows B and D from the same hemisphere was pooled. Samples from different animals were not pooled. In summation, three samples were collected from animals in the conditioning experiment (row B: right hemisphere, row D: right hemisphere, and row B: left hemisphere) and four samples were collected from deprived animals (row C: experimental hemisphere, rows B + D: experimental hemisphere, row C: control hemisphere, and rows B + D: control hemisphere). RNA was recovered using a PicoPure RNA Isolation Kit (KIT0204, Life Technologies) with concurrent genomic DNA elimination using DNase (RNase-Free DNase Set, 79254 Qiagen).

### 2.4. Real-Time PCR

Reversed transcription was performed using a Maxima First Strand cDNA Synthesis Kit (K1641Thermo Scientific-Fermentas). Real-time PCR was conducted using Power SYBR Green PCR Master Mix (4368702, Life Technologies). In real-time PCR experiment, each sample was run in triplicate. Amplification was carried out with a 7500 real-time PCR System (Applied Biosystems), using Power SYBR Green PCR Master Mix, specific primers (Table 1), and cDNA for each sample. The glyceraldehyde 3-phosphate dehydrogenase (GAPDH) gene was used as a housekeeping gene. The amplification reaction was cycled 40 times with a 95°C denaturation step for 15 s and a 60°C annealing step for 1 minute. A dissociation stage was performed to assess specificity of primers. Results were calculated using standard curve method.

### 2.5. Data Analysis and Statistics

Ratios of a target gene and housekeeping gene levels were used for analysis. Statistical analysis was performed using GraphPad Prism 5 software (GraphPad Software, Inc.). Gene expression levels in different barrel rows were analyzed using ANOVA followed by Newman-Keuls post hoc tests. Student's *t*-test was used where applicable.

## 3. Results

### 3.1. Sensory Deprivation

#### 3.1.1. Npas4 and c-Fos

Mice were subjected to one week of sensory deprivation—all vibrissae on one side of the snout were removed except for row C, while vibrissae on the other side were left intact. On the day preceding stimulation, the other side of the snout was subjected to the same procedure, so that mice were left with two C rows intact. Animals were placed in the stimulatory cage for 30 minutes—a time interval demonstrated to be appropriate to observe increase in Npas4 expression in the barrel cortex after exploration of an enriched environment [35]. Regions of interest were microdissected from slices of layer IV. Real-time PCR method was used to assess Npas4 mRNA level in individual barrel rows. Npas4 level was evaluated in spared rows C and in deprived rows B and D in the experimental and control hemisphere.

**Figure 3:**
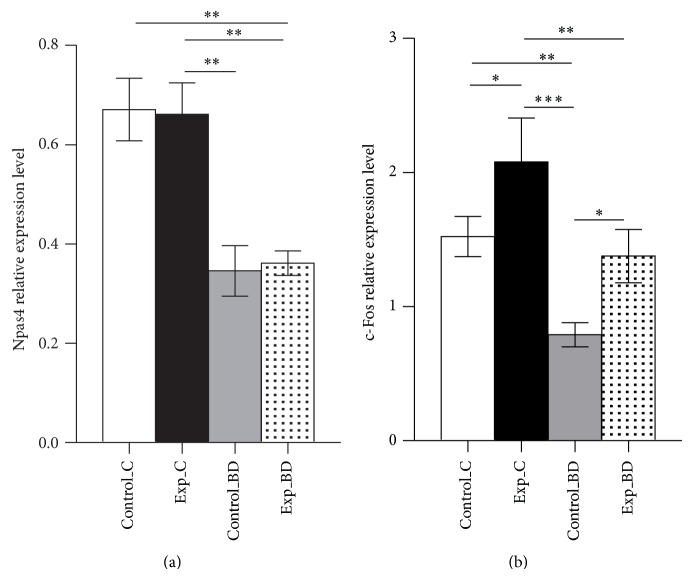
(a) Npas4 expression in spared C rows and in deprived regions (averaged rows B and D). Control_C: spared row C on control side (deprived for 24 hours); Exp_C: spared row C on experimental side (deprived 7 days); Control_BD: deprived B and D rows on control side; Exp_BD: deprived B and D rows on experimental side; mean ± SEM ^**^ *P* < 0.01. Duration of deprivation had no impact on Npas4 expression. (b) In deprived B and D rows deprivation lasting 7 days induced increase in c-Fos expression; mean ± SEM ^*^ *P* < 0.05; ^**^ *P* < 0.01; ^***^ *P* < 0.001.

Elimination of sensory input to selected rows of vibrissae evoked differences in Npas4 expression between spared and deprived barrels (ANOVA, *F*(3.18) = 10.04, *P* = 0.0004, Figure 3(a)). Post hoc analysis demonstrated that the level of Npas4 transcript in the control hemisphere was 48.4% lower in deprived rows B and D than in the spared row C (0.35 ± 0.05 versus 0.67 ± 0.06, *P* < 0.01). There were no differences in Npas4 transcript levels between spared rows C in both hemispheres (*P* > 0.05). Also, no differences were observed between deprived regions in both hemispheres (*P* > 0.05), which shows that duration of deprivation (7 days versus 24 hours) had no impact on Npas4 expression in deprived barrel rows. We have previously shown that this deprivation procedure followed by two hours of exploration of stimulatory cage resulted in an increased number of cells expressing c-Fos in deprived rows B and D after 7 days of deprivation in comparison with 24 hours of deprivation [18]. We also observed this phenomenon regarding the other activity-regulated genes Arc and Zif268 [36]. As Npas4 is also an activity-regulated gene, we expected a similar pattern of its expression. As this was not the case, we decided to assess c-Fos mRNA in the same samples to confirm that the Npas4 pattern of expression is atypical of other activity-regulated genes in this model of plasticity. The deprivation procedure produced differences in c-Fos expression among analyzed barrel rows (ANOVA, *F*(3.16) = 8.028, *P* = 0.0022, Figure 3(b)). In the experimental hemisphere, the level of c-Fos mRNA was 74.2% higher in deprived rows B and D (1.38 ± 0.20 versus 0.79 ± 0.09, *P* < 0.05) than in homotypic regions in the control hemisphere.

### 3.2. Sensory Conditioning

**Figure 4:**
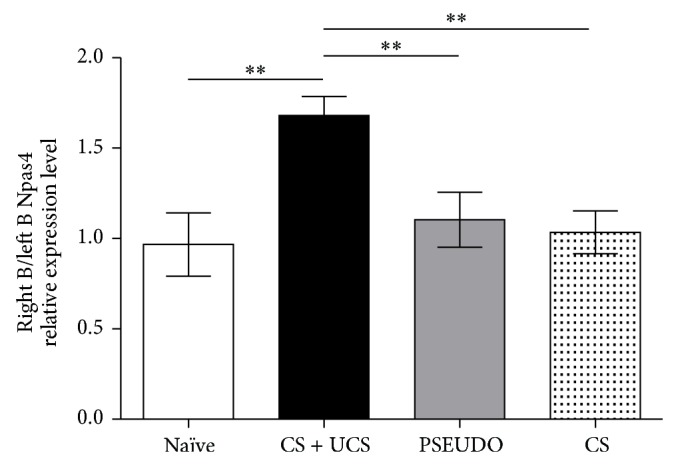
Changes in Npas4 expression induced by training. In trained (CS + UCS) animals expression of Npas4 in the right (trained) row B of barrels is elevated in comparison with left (control, unstimulated) row B. Sole stimulation of whiskers (CS only) and application of unpaired CS and UCS (PSEUDO) do not produce increase in Npas4 expression in comparison with control side. Data are presented as ratio of Npas4 expression level in right and left row B of barrels; mean ± SEM ^**^ *P* < 0.01.

**Figure 5:**
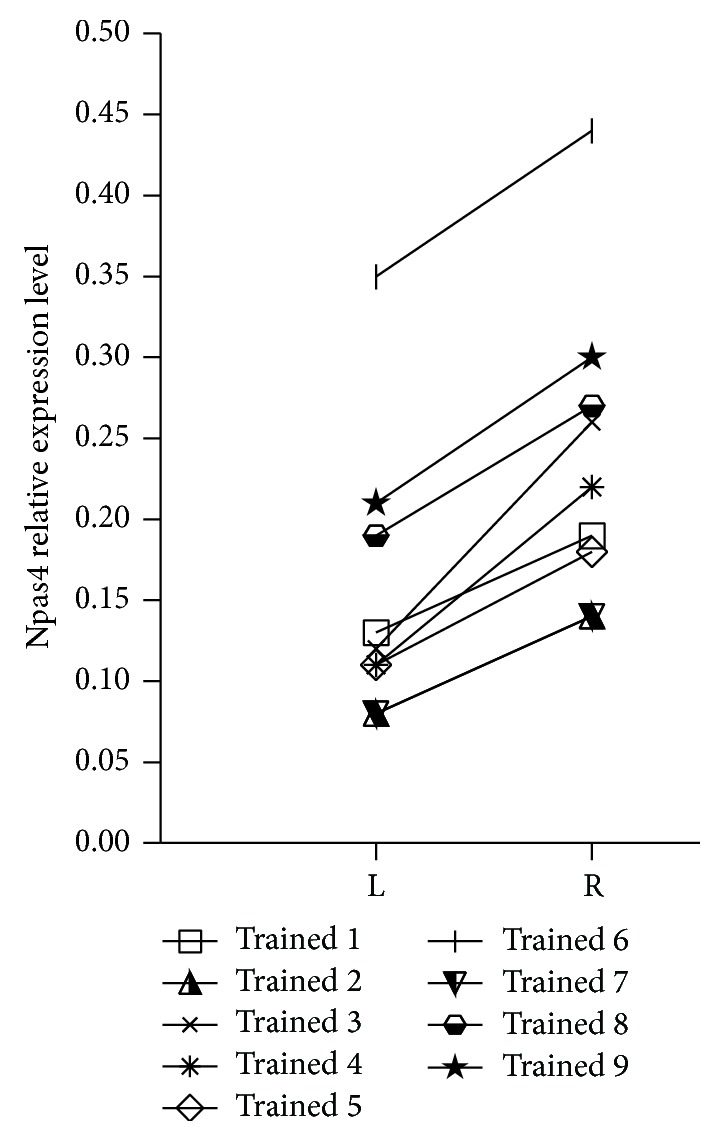
Changes of Npas4 mRNA expression in individual conditioned mice—comparison between trained row B of barrels (R: right) and control row B (L: left).

The training procedure encompassed three training sessions on three consecutive days, each lasting for 10 minutes and consisting of 40 CS (stroking row B of vibrissae) and UCS (electric shock to the tail) pairings. This paradigm evokes freezing-like behavior, which indicates that association of CS and UCS occurred [37]. In CS + UCS animals Npas4 expression was on average 52.9% higher in trained row B barrels than in the contralateral row B (0.24 ± 0.03 versus 0.15 ± 0.03, *t*-test, *P* < 0.001). We found no differences in Npas4 expression between hemispheres in naïve animals, and for further analysis we pooled data from both B rows. Npas4 expression in the trained row B for the CS + UCS group was 48.1% higher than in naïve group B rows (*t*-test, *P* < 0.05). There were no differences between B rows in the naïve group and left (control) row B in the CS + UCS group (*t*-test, *P* > 0.05). Analysis of ratios of Npas4 expression in right row B (trained row in CS + UCS group) and left row B revealed significant effect of the training procedure (ANOVA, *F*(3.25) = 6.780, *P* = 0.0017, Figure 4). Ratio of Npas4 expression in right row B and left row B was higher in CS + UCS group in comparison with other experimental groups (*P* < 0.01), indicating that interhemispheric difference in Npas4 mRNA level is not just an effect of stimulation of the row B of vibrissae. Npas4 expression was increased in the “trained” row in every single conditioned animal (Figure 5).

**Figure 6:**
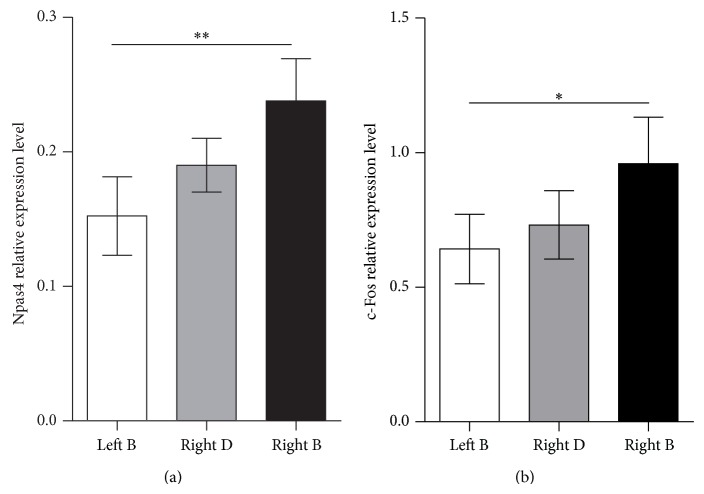
Training induced changes in Npas4 (a) and c-Fos (b) expression. Right B corresponds to the trained row of barrels, right D to control row of barrels in the same hemisphere, and left B to control row B in the other hemisphere. Npas4 and c-Fos have similar pattern of expression following the training procedure: their expression is elevated in the trained row B in comparison with the control side; mean ± SEM ^*^ *P* < 0.05; ^**^ *P* < 0.01.

To see if the observed changes in Npas4 expression are limited to the stimulated row B barrels, we evaluated the level of its expression in row D barrels in the same hemisphere. We did not assess Npas4 mRNA level in row D of the left (control) hemisphere in the CS + UCS group, but taking into account results from the naïve group it can be presumed that it is comparable to the control row B. The level of Npas4 expression in the right row D was an intermediate value between the levels of Npas4 mRNA expression in the right (trained) row B and left (control) row B; it was not significantly different from any of the B rows (Figure 6(a)). It can be interpreted that training affects row D so there are no differences between trained row B and row D in the same hemisphere, but the impact of training upon row D is weak so no differences can be observed when compared to the control hemisphere.

In the deprivation study Npas4 turned out to have a different pattern of expression than other activity-regulated genes and we wondered if it is also the case in sensory conditioning. We evaluated c-Fos mRNA levels in samples obtained from CS + UCS mice. The pattern of c-Fos expression was the same as for Npas4 (Figure 6(b)). The training procedure evoked differences among c-Fos expressions in examined barrel rows (ANOVA, *F*(2,24) = 4.407, *P* = 0.0299). c-Fos mRNA expression level was 48.4% higher in the trained row of barrels (row B in the right hemisphere) than in the contralateral row B (in the left hemisphere, *P* < 0.05).

## 4. Discussion

Knowledge on Npas4 in plasticity of adult cortex is limited. Herein we provide first description of Npas4 expression in the barrel cortex undergoing plastic reorganization in two paradigms: deprivation and sensory conditioning. We also compare changes in expression of Npas4 and c-Fos in both experimental models.

We found that classical conditioning, in which stimulation of a row of whiskers is paired with tail shock, results in increased expression of Npas4 mRNA in the cognate row of cortical barrels. As conditioning triggers inhibitory synaptogenesis in the barrels representing stimulated vibrissae [33], our results are in agreement with data demonstrating a role of Npas4 in the formation of GABAergic synapses. The work of Lin et al. [3] found that Npas4 regulates expression of a variety of genes, including gene coding for BDNF. Npas4 binds to activity-dependent promoters I and IV of the BDNF gene [3], and expression of the BDNF gene from promoter IV contributes to the plasticity of inhibitory synapses [38, 39]. Npas4 expression was demonstrated to drive inhibitory synaptogenesis on excitatory neurons and Npas4 knockdown increases interevent interval and decreases the amplitude of mIPSCs [3]. In the current experiment we observed an increase in Npas4 expression within the trained row, where previously we found increases of GABAergic synapses density, increased synaptic content of GABA, and increased spontaneous IPSCs [33, 40]. Our findings are also in line with the recent results by Sim et al. [4] who found that increased cell intrinsic activity results in, via an Npas4 dependent mechanism, the addition of GABAergic inputs to the neuron. In hippocampal pyramidal neurons behaviorally induced expression of Npas4 drives redistribution of inhibitory synapses, increasing inhibitory synapse number on the cell body while decreasing the number of inhibitory synapses on the apical dendrites [5]. In contrast, in our previous experiments, we observed an increase in density of GABAergic synapses located on spines in barrels representing “trained” vibrissae [33]. This discrepancy could be attributed to possibility that Npas4 impact on dendritic and somatic pool of GABAergic synapses depends on the type of neuron, its location within the brain, and the type of stimulation used to evoke plasticity.

Interestingly, it was recently found that increased Npas4 expression may also account for increased number of excitatory contacts made onto somatostatin interneurons [6]. In the CS + UCS mice an increase in density of somatostatin interneurons within layer 4 of the barrel cortex is observed [41], which may be a result of elevated neuronal activity [42]. It can be hypothesized that Npas4 expression drives generation of additional excitatory input onto somatostatin interneurons, which in turn express higher level of somatostatin.

Herein we demonstrate that sensory conditioning produces increase in Npas4 expression in the region, where inhibitory synaptogenesis was previously observed [33]. Synapses formation and elimination accompanying learning-related behaviors may contribute to shift in excitation-inhibition balance. Dysregulation of this balance has been implicated in number of human neuropsychiatric and neurodegenerative disorders and associated with impairment of cognitive functions [43]. Regarding Npas4 significance for maintaining excitation-inhibition equilibrium, it can be presumed that Npas4 deficiency would result in cognitive deficits. Indeed, such deficits were observed in Npas4 knockout mice [44, 45]. So far Npas4 has not been directly linked to human neuropsychiatric disorders. However, Bersten et al. [46] identified human variants of Npas4 with reduced transcriptional activity, so sequencing Npas4 in neuropsychiatric patients might be helpful in detecting such a link, if it exists.

Previous experiments regarding fear conditioning and contextual learning indicate that Npas4 is indispensable for memory formation [11, 12]. Therefore it is reasonable to think that Npas4 deficiency should also impair sensory conditioning. Testing this hypothesis using Npas4 knockout animals might be misleading: Npas4 knockout animals performed well in amygdala-dependent fear conditioning [11], while acute deletion of Npas4 in amygdala impaired fear memory formation [12]. Regionally selective depletion of Npas4 might be more useful then, but first structures required for learning association of sensory CS and UCS in paradigm used in this study should be identified. Basolateral amygdala is activated during the training procedure [37] and amygdala is required for all forms of fear conditioning [47]; therefore amygdala would seem a first choice structure for local knockdown of Npas4 and determining its impact on sensory conditioning.

Npas4 expression in remote row D barrels did not differ from the results obtained for the control hemisphere and naïve group, which is in agreement with our previous observation that no changes typical for the trained row B appear in remote row D (no AMPA and NMDA binding increase, no GAD mRNA expression upregulation, and no increase in density of somatostatin containing inhibitory interneurons) [40, 41, 48, 49]. We did not do EM to evaluate the synapse density of remote row D barrels, but we found no increase in spontaneous IPSC there, which would indicate increased GABA release in its excitatory neurons [40].

In the sensory conditioning paradigm Npas4 and c-Fos levels changed in the same way. They were upregulated in the row of barrels that received the conditioned input.

Depriving selected barrel rows of sensory input resulted in decreased Npas4 mRNA expression in the deprived barrel rows in comparison with spared rows. Duration of deprivation (24 h versus 7 days) had no impact upon Npas4 expression in spared rows and deprived rows. This contrasted with c-Fos, the expression of which was increased in both deprived and spared regions after 7 days of deprivation in comparison with 24 hours of deprivation.

Using immunohistochemical techniques, we previously described the pattern of c-Fos protein expression following whisker deprivation of various durations [18] in the same deprivation paradigm. The density of c-Fos positive nuclei increased in the barrel row deprived of whisker input for 7 days, and the effect was augmented as the deprivation period was prolonged. We interpreted this result as illustrating the expansion of the spared vibrissal input into neighboring, functionally deafferented barrels. The gradual increase in the number of immunoreactive nuclei could reflect the gradual rewiring of the barrel cortex in the course of prolonged deprivation. The present results confirm our previous data at the mRNA level.

Unlike c-Fos, Npas4 mRNA in the deprived B and D rows was not upregulated by sensory stimulation when comparing 24 h versus 7 days of deprivation. We suppose that the strength of the sensory signal coming from the spared row C whiskers was insufficient for changing the expression of this IEG. In the experimental paradigm used here we previously observed pronounced changes in Zif268 and Arc expression [36]. It is not surprising that differently regulated genes do not respond identically to an experimental situation (see [35] for comparison of several IEGs activations by enriched environment). Perhaps the plastic rearrangement of connections induced by deprivation, although it already changes the metabolic response [18], does not yet trigger activation in Npas4 mRNA expression. Npas4 can regulate activity-dependent expression of Arc, c-Fos, and Zif268 [11]. Taking into consideration that Arc, c-Fos, and Zif268 expressions increase in deprived barrels after 7 days of deprivation, it may seem puzzling that expression of Npas4, which regulates the transcription of these genes, remains unaltered. However, it should be noted that Npas4 itself is an immediate early gene and gets activated in response to stimulation. Accordingly, it probably does not regulate the first phase of other immediate early genes expression, which is independent of* de novo* protein synthesis. It rather seems that Npas4 plays a role in enhancing and sustaining other immediate early genes in later phases [11].

To the best of our knowledge this is the first report on Npas4 expression in deprivation-induced plasticity. Maya-Vetencourt et al. [13] examined Npas4 expression in monocularly deprived rats treated with fluoxetine, but they concentrated on influence of fluoxetine on Npas4 expression and not on the influence of deprivation.

In this paper the precise anatomical dissection of a row of barrels where a plastic change took place allowed for a quantitative analysis of IEGs expression in two types of experience dependent plasticity. Activation of the barrel cortex undergoing reorganization triggered by removal of selected rows of whiskers strongly affected c-Fos (but not Npas4) expression. Activation of the cortex undergoing a plastic change due to being involved in sensory conditioning upregulated the expression of both Npas4 and c-Fos. Taking into consideration our observations that sensory conditioning increases the number of inhibitory synapses within the trained barrels and studies of other groups showing involvement of Npas4 in synaptogenesis, we presume that Npas4 may be involved in reshaping of connectivity within barrel cortex after sensory conditioning.

## Figures and Tables

**Table 1:** Sequences of primers used in real-time PCR.

Target gene	Primers sequence (5′ → 3′)
Npas4 (NM_153553.4)	Forward: TGCTGGAGGCACTCCTTTGGC
	Reverse: GCTGCTGGCGCACAGTGAGA

c-Fos (NM_010234.2)	Forward: CGGGTTTCAACGCCGACTA
	Reverse: TTGGCACTAGAGACGGACAGA

GAPDH (NM_008084.2)	Forward: CGGCAAATTCAACGGCACAGTCAA
	Reverse: TGGGGGCATCGGCAGAAGG
